# Supplementary material for: Fucoxanthin Prevents Pancreatic Tumorigenesis in C57BL/6J Mice That Received Allogenic and Orthotopic Transplants of Cancer Cells
Source: Int J Mol Sci. 2021 Dec 19;22(24):13620. doi: 10.3390/ijms222413620 (PMC8707761; doi:10.3390/ijms222413620)
Supplement: Supplementary file 1 [file ijms-22-13620-s001.zip › ijms-1483444-supplementary.pdf]

International Journal of *Molecular Sciences*

MDPI

Supplementary Table S1. Profile of upregulated genes (< 2.0 and ≥ 1.5-fold change) in pancreatic tumors of mice treated with fucoxanthin (Fx).[1]

Gene symbol	Description	Fold change [2]	*p*-value [3]
Mlph	Melanophilin	1.99	0.0014
Apln	Apelin	1.98	0.0298
Trim30c	Tripartite motif-containing 30C	1.97	0.0073
Trem1	Triggering receptor expressed on myeloid cells 1	1.94	0.0116
Elf3	E74-like factor 3	1.84	0.0011
Fancg	Fanconi anemia, complementation group G	1.80	0.0023
Fam3b	Family with sequence similarity 3, member B	1.79	0.0333
Gpr84	G protein-coupled receptor 84	1.78	0.043
Cd55b	CD55 molecule, decay accelerating factor for complement B	1.77	0.0023
Arl14epl	ADP-ribosylation factor-like 14 effector protein-like	1.77	0.0145
Dmd	Dystrophin, muscular dystrophy	1.76	0.0328
Tnfrsf21	Tumor necrosis factor receptor superfamily, member 21	1.76	0.0066
Hydin	HYDIN, axonemal central pair apparatus protein	1.76	0.0119
Rorc	RAR-related orphan receptor gamma	1.74	0.0418
Il24	Interleukin 24	1.73	0.007
Per1	Period circadian clock 1	1.73	0.001
Trim13	Tripartite motif-containing 13	1.70	0.0188
Tcaim	T cell activation inhibitor, mitochondrial	1.70	0.027
Olfr6	Olfactory receptor 6	1.69	0.0339
Olfr1277	Olfactory receptor 1277	1.69	0.0381
Leng9	Leukocyte receptor cluster (LRC) member 9	1.68	0.0155
Adam8	A disintegrin and metallopeptidase domain 8	1.68	0.0367
Slc35f1	Solute carrier family 35, member F1	1.68	0.0054
F10	Coagulation factor X	1.66	0.0069
Defb8	Defensin beta 8	1.66	0.0099
Fzd5	Frizzled homolog 5 (Drosophila)	1.66	0.0046
Nlrp10	NLR family, pyrin domain containing 10	1.65	0.0148
Hspa1b; Hspa1a	Heat shock protein 1B; heat shock protein 1A	1.65	0.0234
Cwh43	Cell wall biogenesis 43 C-terminal homolog (S. cerevisiae)	1.64	0.0226
Cxcl5	Chemokine (C-X-C motif) ligand 5	1.64	0.0397
Olfr640	Olfactory receptor 640	1.63	0.0041
Crisp3	Cysteine-rich secretory protein 3	1.63	0.0007
BC100530; Stfa3	cDNA sequence BC100530; stefin A3	1.63	0.0203
Kctd1	Potassium channel tetramerisation domain containing 1	1.63	0.0076
Osm	Oncostatin M	1.62	0.0225
Car2	Carbonic anhydrase 2	1.62	0.004
Ms4a13	Membrane-spanning 4-domains, subfamily A, member 13	1.61	0.0114
Hsph1	Heat shock 105kDa/110kDa protein 1	1.61	0.0254
Igsf9b	Immunoglobulin superfamily, member 9B	1.60	0.0176

Gnrh1	Gonadotropin releasing hormone 1	1.59	0.0081
Aqp7	Aquaporin 7	1.59	0.0114
Spink8	Serine peptidase inhibitor, Kazal type 8	1.58	0.0339
Bhlhe40	Basic helix-loop-helix family, member e40	1.57	0.0248
Bche	Butyrylcholinesterase	1.57	0.0308
Prrg3	Proline rich Gla (G-carboxyglutamic acid) 3 (transmembrane)	1.55	0.005
Banp	BTG3 associated nuclear protein	1.54	0.0383
Cdhr2	Cadherin-related family member 2	1.54	0.0074
Ppp2r4	Protein phosphatase 2A activator, regulatory subunit B	1.54	0.0031
Slc29a2	Solute carrier family 29 (nucleoside transporters), member 2	1.54	0.0046
Tcirg1	T cell, immune regulator 1, ATPase, H+ transporting, lysosomal V0 protein A3	1.54	0.0248
4933417A18Rik	RIKEN cDNA 4933417A18 gene	1.54	0.0478
Zbtb40	Zinc finger and BTB domain containing 40	1.53	0.0154
Zfp945	Zinc finger protein 945	1.53	0.0012
Serpinb5	Serine (or cysteine) peptidase inhibitor, clade B, member 5	1.52	0.0424
Hspa1a	Heat shock protein 1A	1.52	0.0226
Sord	Sorbitol dehydrogenase	1.51	0.049
Slc4a2	Solute carrier family 4 (anion exchanger), member 2	1.51	0.0435
Vmn1r100	Vomeronasal 1 receptor 100	1.51	0.0063
Vmn1r148	Vomeronasal 1 receptor 148	1.51	0.0063
4930549C01Rik	RIKEN cDNA 4930549C01 gene	1.51	0.0157
Olfr191	Olfactory receptor 191	1.50	0.004

[1] Among 86 upregulated genes, 61 upregulated genes with < 2.0 and ≥ 1.5-fold change were showed. [2] Fold change in gene expression in pancreatic tumors of mice with Fx administration (group 1) compared to that of control mice (group 2). [3] Significant difference between groups 1 and 2 by an exact test on edge R.

Supplementary Table S2. Profile of downregulated genes (> -2.0 and < -1.5-fold change) in pancreatic tumors of mice treated with fucoxanthin (Fx).[1]

Gene symbol	Description	Fold change [2]	*p*-value [3]
Trpv6	transient receptor potential cation channel, subfamily V, member 6	-1.98	0.0003
Gpr183	G protein-coupled receptor 183	-1.95	0.0046
Itih5	inter-alpha (globulin) inhibitor H5	-1.94	0.0427
Dock10	dedicator of cytokinesis 10	-1.94	0.0207
Ces2b	carboxyesterase 2B	-1.91	0.0383
Zc3h12d	zinc finger CCCH type containing 12D	-1.86	0.044
Fam205a2; Gm10600	family with sequence similarity 205, member A2; predicted gene 10600	-1.83	0.0098
Slc7a14	solute carrier family 7 (cationic amino acid transporter, y+ system), member 14	-1.82	0.0147
Sytl5	synaptotagmin-like 5	-1.77	0.0053
Stra6l	STRA6-like	-1.76	0.0096
Gimap9	GTPase, IMAP family member 9	-1.73	0.0152
Il22ra1	interleukin 22 receptor, alpha 1	-1.71	0.0419
Ackr4	atypical chemokine receptor 4	-1.69	0.0106
Nov	nephroblastoma overexpressed gene	-1.68	0.0371
Fetub	fetuin beta	-1.68	0.005
Gnao1	guanine nucleotide binding protein, alpha O	-1.67	0.0061
Kcnh5	potassium voltage-gated channel, subfamily H (eag-related), member 5	-1.67	0.0424
Vnn3	vanin 3	-1.66	0.0036
St6galnac2	ST6 (alpha-N-acetyl-neuraminyl-2,3-beta-galactosyl-1,3)-N-acetylgalactosaminide alpha-2,6-sialyltransferase 2	-1.66	0.0006
Aldh1a7	aldehyde dehydrogenase family 1, subfamily A7	-1.66	0.0136
Far2	fatty acyl CoA reductase 2	-1.66	0.0426
Trmt11	tRNA methyltransferase 11	-1.65	0.0433
Mmp16	matrix metallopeptidase 16	-1.65	0.0055
Gulo	gulonolactone (L-) oxidase	-1.64	0.028
Fmo1	flavin containing monooxygenase 1	-1.63	0.0065
Pkib	protein kinase inhibitor beta, cAMP dependent, testis specific	-1.63	0.0081
Klk9	kallikrein related-peptidase 9	-1.63	0.0035
Cd79a	CD79A antigen (immunoglobulin-associated alpha)	-1.62	0.0231
Olfr1058	olfactory receptor 1058	-1.62	0.0129
Slc7a11	solute carrier family 7 (cationic amino acid transporter, y+ system), member 11	-1.62	0.0307
Ppp4r4	protein phosphatase 4, regulatory subunit 4	-1.61	0.0448
Vmn2r44	vomeronasal 2, receptor 44	-1.61	0.0389
Uts2	urotensin 2	-1.60	0.0252
Cygb	cytoglobin	-1.58	0.026

Sptbn2	spectrin beta, non-erythrocytic 2	-1.58	0.0212
Slc4a8	solute carrier family 4 (anion exchanger), member 8	-1.57	0.0097
Slc35c2	solute carrier family 35, member C2	-1.57	0.0126
Olfr610	olfactory receptor 610	-1.56	0.0178
Crb1	crumbs family member 1, photoreceptor morphogenesis associated	-1.56	0.0013
Gng11	guanine nucleotide binding protein (G protein), gamma 11	-1.56	0.0212
Sema6a	sema domain, transmembrane domain (TM), and cytoplasmic domain, (semaphorin) 6A	-1.55	0.0262
Cyp3a59	cytochrome P450, family 3, subfamily a, polypeptide 59	-1.55	0.0107
Enkur	enkurin, TRPC channel interacting protein	-1.54	0.0421
Hdgfrp3	hepatoma-derived growth factor, related protein 3	-1.54	0.0032
Siglecg	sialic acid binding Ig-like lectin G	-1.53	0.0307
Fam161a	family with sequence similarity 161, member A	-1.52	0.0042
Plk2	polo-like kinase 2	-1.52	0.0394
Olfr44	olfactory receptor 44	-1.51	0.0304
Olfr1247	olfactory receptor 1247	-1.51	0.0023
Abca8a	ATP-binding cassette, sub-family A (ABC1), member 8a	-1.50	0.0307
Setd4	SET domain containing 4	-1.50	0.0348
5031410I06Rik	RIKEN cDNA 5031410I06 gene	-1.50	0.0192

[1] Among all 88 downregulated genes significantly altered, 52 downregulated genes with > -2.0 and < -1.5-fold change were showed. [2] Fold change in gene expression in pancreatic tumors of mice with Fx administration (group 1) compared to that of control mice (group 2). [3] Significant difference between groups 1 and 2 by an exact test on edge R.
